# Peristaltic infusion of red blood cells: analysis of hemolysis ratio, plasma hemoglobin and potassium levels

**DOI:** 10.1186/2197-425X-3-S1-A921

**Published:** 2015-10-01

**Authors:** M Pedreira, MA Peterlini, AM Avelar, DM Kusahara

**Affiliations:** Federal University of São Paulo, Pediatric Nursing, São Paulo, Brazil

## Introduction

It is unclear whether the use of linear peristaltic infusion pumps with different propulsion mechanisms can affect the quality of red blood cells (RBCs) transfusion. We hypothesized that the mechanism of liquid propulsion, infusion rate, complete or incomplete compression of the infusion sets segment and type of pumping segment composition materials are variables that can influence RBC infusion quality.

## Objectives

To analyze the effects of two linear peristaltic infusion pumps on RBC hemolysis ratio, plasma hemoglobin and potassium levels, according to the use two different infusion rates.

## Methods

Experimental transfusions of RBC simulating nursing clinical practice were accomplished in three linear peristaltic infusion pumps with horizontal peristaltic finger mechanism (A) and three linear peristaltic infusion pumps with a vertical peristaltic mechanism that compress the administration tube halfway to closure (B). The infusion pumps were set at 100 ml/h and 300 ml/h. The six pumps and the two infusion rates were randomly submitted to analysis. Plasma hemoglobin (g/dl), hemolysis ratio (%) and potassium levels (mmol/L) were assessed in RBC before manipulation directly from the RBC bag (C1), after free flow through the macro drip intravenous infusion sets (C2) and after infusion by the peristaltic mechanisms (E). Data were analyzed according to mean, standard deviation, and t test, significance level set as p≤ 0.05.

## Results

A significant effect of the pump mechanism was verified between infusion pumps A and B only in plasma hemoglobin (A = 0.419 ± 0.216; B = 0.251 ± 0.205; p = 0.021). A statically similar plasma potassium level (A=39.6 ± 3.0; B=40.3 ± 2.3; p = 0.471) and hemolysis ratio (A = 0.582 ± 0.256; B = 0.392 ± 0.404; p = 0.089) were obtained. Hemolysis between C1 to C2 and E presented variation in infusion pump A ranging from 0.503(± 0.740) in C1, 0.417(± 0.610) in C2 and to 1.131(± 1.180) in E. According to the studied infusion rates, hemolysis was higher to infusion pump A at 100 ml/h (p < 0.0001) and infusion pump B at 300 ml/h (p = 0.004). The plasma hemoglobin level was higher in infusion pumps A at 100 ml/h (p = 0.009) and similar between pumps (p = 0.576) at 300 ml/h. The plasma potassium level variation according to infusion rates did not present significant variation (p > 0.05).

## Conclusions

Comparisons between pumps demonstrated a higher increment of plasma hemoglobin in infusion pump A. According to the studied rates, hemolysis ratio was higher in infusion pump A at 100 ml/h and in infusion pump B at 300 ml/h; plasma hemoglobin was higher in infusion pump A at 100 ml/h; plasma potassium levels had no significant variations. A hemolysis ratio higher than 0.8% was verified only in infusion pump A at 100 ml/h.

## Grant Acknowledgment

FAPESP-Sao Paulo Research Foundation n.12/25284-9.

